# Virtual Human Role Players for Studying Social Factors in Organizational Decision Making

**DOI:** 10.3389/fpsyg.2018.00194

**Published:** 2018-03-01

**Authors:** Peter Khooshabeh, Gale Lucas

**Affiliations:** ^1^US Army Research Laboratory, Human Research and Engineering Directorate, Los Angeles, CA, United States; ^2^Institute for Creative Technologies, University of Southern California, Los Angeles, CA, United States

**Keywords:** social influence, persuasion, virtual humans, emotion, training

## Abstract

The cyber domain of military operations presents many challenges. A unique element is the social dynamic between cyber operators and their leadership because of the novel subject matter expertise involved in conducting technical cyber tasks, so there will be situations where senior leaders might have much less domain knowledge or no experience at all relative to the warfighters who report to them. Nonetheless, it will be important for junior cyber operators to convey convincing information relevant to a mission in order to persuade or influence a leader to make informed decisions. The power dynamic will make it difficult for the junior cyber operator to successfully influence a higher ranking leader. Here we present a perspective with a sketch for research paradigm(s) to study how different factors (normative vs. informational social influence, degree of transparency, and perceived appropriateness of making suggestions) might interact with differential social power dynamics of individuals in cyber decision-making contexts. Finally, we contextualize this theoretical perspective for the research paradigms in viable training technologies.

## 1. Introduction

As new skills are required for the twenty first century global enterprise, there are conceivable situations where a new employee to an organization could have vastly greater technical capabilities than the supervisor to whom she reports. Here the new employee must tread carefully because although she has more technical knowledge than her superior, she still lacks the organizational power to be able to influence any decisions by her leadership. In this article, we focus on how this social dynamic is relevant to behavioral cyber science. Specifically, the scientific literature in the fields of social influence and persuasion can provide clues how to manage this delicate dynamic. So the article begins with a delineation of relevant psychological factors that can affect the human dimension in cyber science. Next, we propose potential training techniques to address psychological challenges that could impede cyber power. We conclude with next steps for future research.

## 2. Cognitive and socio-cultural factors in cyber science

Cognitive and social psychological factors have been reported to play a potential role in cyber operations. In a briefing to the US Army Special Operations Command (USASOC) open Futures Forum, Schneider (2017) reported on a longitudinal study that used a series of war games to study whether cyber effects increase the chance of inadvertent conflict. The focus of the war games was on crisis decision making, not necessarily cyber, and they took place at the Naval War College over 2011–2016. Across that time frame, a general result was that the war gamers were more comfortable using conventional kinetic force, even a nuclear standby, than taking cyber defensive operations, including cyber information operations. Schneider gives a few possible interpretations that could explain this risk aversion toward cyber operations. First, there might be a general perception that the United States is more vulnerable to cyber attacks than its adversaries; hence, this might breed a reluctance and uncertainty to use cyber effects in fear of retaliation and escalation. Related to this, uncertainty could arise because of concerns about the domestic implications of cyber attacks on adversary (civilian) populations. A second factor driving the uncertainty could be concerns regarding the relationship between cyber and nuclear operations. Finally, a cognitive and affective science explanation could be that cyberspace is processed differently than other evolutionary (physical) threats that relate to conditioning of threat/fear (Mutlu, 2017). Because cyber requires some context, it produces anxiety that in turn causes inaction (Roese et al., 1999). In addition to the cognitive factors related to uncertainty, there are socio-cultural human dimensions in the cyber domain. Although there is not much research on the social and cultural characteristics of cyber operators, recent case studies help begin to paint a picture. Based on interviews of cadets in the Norwegian Defence Cyber Academy, Roislien (2015) reports on the generational gap between junior personnel compared to higher-ranking officers in the cyber domain. This gap could be potentially problematic because the younger cadets have essentially been brought up in a contemporary (Western) context where technology is taken for granted, the cultural ethos focuses on self-expression, and thinking is dominated by intuitive emotional cues. As these digital natives are indoctrinated in the military hierarchical system, they have to learn to adapt their way of expressive communication in a more appropriate manner so as to influence decision making of senior leaders on complex cyber issues. Below we discuss potential techniques for junior cyber operators, and in some cases their senior officers, to improve such communication.

### 2.1. Type of social influence

Research in social science distinguishes between two types of social influence: informational and normative (Cialdini and Goldstein, 2004). Informational social influence is driven by the desire to evaluate ambiguous situations correctly, whereas normative social influence is driven by the desire to be liked and gain social acceptance from another person. The type of social influence might interact with differential social power dynamics of individuals in cyber decision-making contexts. For example, in a recent study, Artstein et al. (2018) compared how users interact with a system that persuades them either using informational or normative social influence, and users were told that the system was either tele-operated by a human (Wizard-of-Oz) or fully-automated (AI). Using this design, Artstein et al. were able to compare the effectiveness of virtual agents (vs. humans) in employing informational vs. normative social influence. Participants interacted with the system, which employed a virtual agent that tried to persuade the user to agree with its rankings on a “survival task” (Artstein et al., 2017). Controlling for initial divergence in rankings between user and the agent, there was a significant main effect such that informational social influence resulted in greater influence than normative influence. However, this was qualified by an interaction that approached significance; users were, if anything, more persuaded by informational influence when they believed the agent was AI (compared to a human), whereas there was no difference between AI and human framing in the normative influence condition. Because AI are often perceived as lower power (as we discuss further below; see Wang et al., 2015), this finding may represent an advantage of informational social influence, especially when the interlocutor has lower power than the agent. It is possible, then, junior cyber operators should engage in informational social influence (more than normative) so as to persuade a leader to make informed decisions.

### 2.2. Transparency to promote more effective communication

Researchers have identified three components that promote trust and influence: benevolence, competence, and integrity (Mayer et al., 1995; Lee and See, 2004; Hoff and Bashir, 2015). With humans and technology alike, we decide whether we can trust another based on: the extent to which their intentions match our goals (benevolence), how well they can execute their intentions (competence), and the extent to which they adhere to a set of acceptable principles (integrity). Most junior cyber operators will intuitively attempt to convey their competence, and shared, good intentions are usually presumed within military teams due to their shared mission. In contrast, the latter factor (integrity) might be overlooked by junior cyber operators as a way to help persuade their leaders to take a particular course of action and overcome social rifts caused by the generational gap (Roislien, 2015). Indeed, research with AI -again- suggests this could be leveraged to help these cyber operators. For example, systems that are more transparent about how they work and how they came to their conclusion—or “explainable AI” (Chen et al., 2014; Gunning, 2017)—have been shown to garner greater trust and influence. Likewise, this finding may suggest an advantage of transparency in cyber decision-making contexts; junior cyber operators may find a benefit of being more transparent in how they come to their conclusions in attempting to persuade leaders to make better decisions. This relates to the *Orienting, Locating, Bridging (OLB)* framework proposed by Knox et al. (in revision) that is intended to train communication skills in cyber defence academies. In this case, transparency can be one way of orienting effectively by conveying implicit metacognitive awareness factors that affect momentary cognitive processes and mental states.

### 2.3. Perceived appropriateness of interpersonal expressions

As the interviews by Roislien (2015) suggest, the current generation of cyber operators in the making are more apt to be emotionally expressive and this might impact the extent to which they are able to influence more senior leaders. Just as there are potentially different paths to social influence, e.g., normative vs. informational, there are multiple interpersonal strategies to influence others through non-verbal displays. For example, it is possible to use emotional facial expressions in order to convey social information in dyadic or group decision-making tasks. Our own previous work has investigated the effects of emotional expressivity when the person observing the emotional expressions of a counterpart was either in a lower or higher ranking power position (Wang et al., 2015). Participants completed rankings in a survival task, similar to Artstein et al. (2017), Khooshabeh et al. (2011), and Wang et al. (2013), where they had the opportunity to discuss their original rankings with an ostensible partner, which was a photorealistic virtual character. In the first study, participants were told that they were in charge and would be interacting with a partner who had a lower rank than them (follower). After making initial decisions about the priority of items most necessary for survival, participants were required to ask at least three questions about their less powerful partner's choices and in turn discuss their own rankings. The less powerful partner then displayed emotional facial expressions that followed a carrot/stick policy where if the participant's choice of item prioritization was similar, then the partner displayed a happy emotional expression; if the participant's choice was not close, then the partner displayed anger. As predicted, Study 1 results showed that participants were less persuaded by the emotionally expressive follower compared to a neutral follower that did not display any facial emotions (Wang et al., 2015).

Based on previous research (Van Kleef and Côté, 2007; Van Kleef et al., 2011), in Wang et al. (2015) we hypothesized that the reason leader participants were not persuaded by the expressive follower could be because emotional expressions were perceived as inappropriate in this context. So Study 2 manipulated whether emotional expressions were perceived to be in/appropriate and whether the individual expressing emotion was a follower or leader. Results showed that followers were more persuaded than leaders, which suggests that simply telling people that they are in a particular role makes them reluctant to accept a lower ranking person's recommendations. Moreover, there was a replication of the finding from Study 1 that emotionally expressive followers were less persuasive than neutral followers. There were also two interaction effects. For the interaction of power and emotion, expressiveness reduced persuasiveness of followers but did not affect leader persuasion. For the interaction of emotion and perceived appropriateness, emotional expressions did not reduce persuasion when they were displayed in an ostensibly appropriate context. Both studies reported in Wang et al. (2015) provide evidence which suggests that individuals in higher ranking positions do not take into account task-relevant information from subservient people who are emotionally expressive, and that social contexts where the expression of emotion is more appropriate, regardless of position on the power spectrum, are more conducive to efficient information exchange.

One implication of these results is that leaders seem to be biased against followers who display emotion. So one potential way to overcome these biases is either to train leaders to become more accepting of followers' emotional expressions or to train followers to regulate their emotional expressions so that they may be more effective at persuading leaders with important information.

## 3. Potential benefits of using virtual human role players

Virtual Humans (VH) role players have been found effective at teaching interpersonal skills such as negotiation and public speaking (Core et al., 2006; Batrinca et al., 2013). Accordingly, there is also potential for VHs to be useful in helping train interpersonal skills in cyber decision-making and teaming contexts. VHs could be useful to help junior cyber operators practice providing convincing information relevant to a mission in order to persuade or influence a leader to make informed decisions. Likewise, leaders could use VHs to practice receiving such information and integrating it to make informed decisions. Research on VHs for interpersonal skills training has shown potential benefits beyond reduced costs and greater ease of dissemination and extensiblity. For example, VH role players have been shown to make people practicing interpersonal skills feel more comfortable than human role players (Gratch et al., 2016). Such benefits could be useful in the cyber decision-making context as well, especially when practicing uncomfortable conversations across different levels of power and technical knowledge (e.g., junior operator to non-technical leader).

Specifically, VH role players could be used to teach junior cyber operators to adapt their way of expressive communication in a more appropriate manner in the three ways described above. They could practice using these techniques to better influence decision making of senior leaders on complex cyber issues. Junior cyber operators can practice engaging in informational social influence with a “virtual leader” so as to gain experience effectively persuading a leader to make informed decisions. Likewise, they could also practice being more transparent in how they come to their conclusions when attempting to persuade these virtual leaders. The virtual leader AI could also be developed to provide feedback or to act like it had been persuaded when operators utilized the appropriate level of transparency. Finally, virtual applications could be beneficial for training cyber operators to regulate their emotional expressions when attempting to persuade leaders with important information. With possible sensory platforms, such as using computer vision to track user's emotional expression or relevant wearable physiological sensors to infer user states, the AI system could provide feedback about how effectively the operator was able to regulate her emotions.

## 4. Discussion and future work

**Figure 1:**
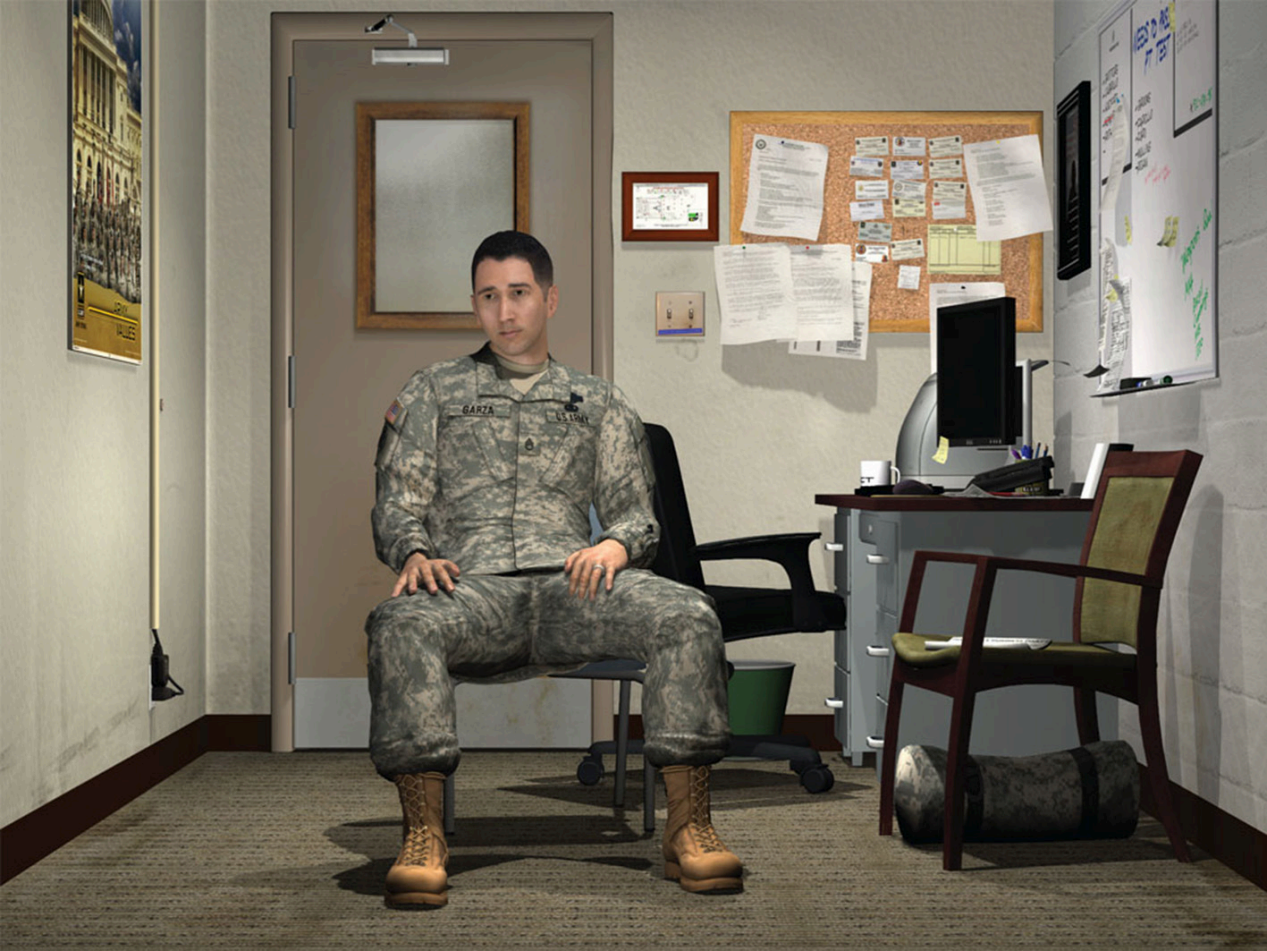
The Emergent Leader Immersive Training Environment (ELITE) is an example of a system that helps develop junior officer communication skills; image reproduced with permission from USC ICT (http://ict.usc.edu/prototypes/elite/).

Advanced virtual human technology has been successfully integrated within various cross-agency military training contexts, including practicing leadership and counseling skills (Hays et al., 2012; Core et al., 2016). The Virtual Human Toolkit (Hartholt et al., 2013) makes it possible to design and develop the technical aspects that help realize these virtual human scenarios (see Figure 1). However, a necessary first step is to conduct and understand cognitive task analyses of the cyber domain that requires social skills training, which should be grounded in the behavioral sciences with respect to both theoretical and empirical foundations. In this article we have surveyed three potential social factors that could play a role in understanding the human dimension of cyber decision making. Informational social influence could be a viable path for junior cyber operators to persuade senior leaders to make effective decisions. An important consideration for junior and senior cyber operators is to consider the appropriateness of naturally occurring emotional expressions because this has been shown to affect the degree to which technically proficient junior personnel are able to persuade leaders. Taken together, having situational awareness about which type of influence to use, conveying transparency of technical knowledge, and perceiving appropriateness of interpersonal expressions can contribute to effective information exchange in junior/senior cyber operators social interactions. Future work should include longitudinal training effectivness studies as well as *in-situ* data collection to understand the theoretical cognitive mechanisms involved in cyber power.

## Author contributions

PK contributed the conception and wrote sections of the manuscript. GL wrote sections of the manuscript.

### Conflict of interest statement

The authors declare that the research was conducted in the absence of any commercial or financial relationships that could be construed as a potential conflict of interest.
